# Side Lighting of Red, Blue and Green Spectral Combinations Altered the Growth, Yield and Quality of Lettuce (*Lactuca sativa* L. cv. “Yidali”) in Plant Factory

**DOI:** 10.3390/plants12244147

**Published:** 2023-12-13

**Authors:** Ren Chen, Zhenwei Wang, Wenke Liu, Yuteng Ding, Qishuan Zhang, Shurong Wang

**Affiliations:** 1 Guangdong-Hong Kong-Macao Joint Laboratory for Intelligent Micro-Nano Optoelectronic Technology, School of Physics and Optoelectronic Engineering, Foshan University, Foshan 528225, China; chenren@fosu.edu.cn (R.C.); wangzw001@foxmail.com (Z.W.); dyt2283912605@163.com (Y.D.); zqsnumber1@163.com (Q.Z.); 2 Institute of Environment and Sustainable Development in Agriculture, Chinese Academy of Agricultural Sciences, Beijing 100081, China; 3 Key Lab of Energy Conservation and Waste Management of Agricultural Structures, Ministry of Agriculture and Rural Affairs, Beijing 100081, China

**Keywords:** plant factory, side lighting, light spectral quality, yield, nutritional quality

## Abstract

A plant factory with artificial lighting (PFAL) usually uses top lighting for cultivation. The light from the upper part of the canopy cannot penetrate the entire lettuce canopy, however, resulting in uneven vertical spatial light in the canopy, and accelerating the senescence of both the bottom and side leaves of the plant canopy. Therefore, in this study, the performance of lettuce in hydroponics was investigated upon supplemental side lighting with different spectral LEDs in a PFAL. A set of short-term side lighting treatments, including no side lamps (CK), red (R), blue (B), red + blue (RB), and red + blue + green (RGB) LED lamps (150 μmol·m^−2^·s^−1^, respectively), was employed for an additional 2 h per day after normal top lighting for 6 days before harvest. The results showed that the lettuce canopy was relatively loose and had a large crown size under side lighting compared with CK. Side lighting, irrespective of spectral qualities, significantly increased the fresh weight, and the R, B, RB, and RGB treatments increased the shoot fresh weight of lettuce plants by 34%, 19%, 31%, and 34%, and increased the fresh weight of leaf layer 2 by 50%, 17%, 44%, and 48%, respectively. The side lighting of different spectral qualities had a significant impact on the nutritional quality of the first row of lettuce at the edge of the top lighting illuminated area. Treatment B significantly promoted the chlorophyll content of leaf layer 3; the soluble sugar contents from leaf layer 1, 2, and 3; the starch contents in leaf layers 2 and 3; and the content of phenolics in the leaf layers 3; and significantly reduced the nitrate content in leaf layers 2 and 3. RGB significantly increased soluble sugar content by 91%, and the starch content in leaf layer 1, as well as the leaf chlorophyll and flavonoid content of leaf layer 3, while R had opposite effect completely. RB significantly increased the leaf chlorophyll content of leaf layer 3 and the nitrate content in leaf layer 1, but the overall effect was lower than that of RGB. In summary, side lighting of any type could effectively improve lettuce yield, solve the problem of inconsistent lettuce plant size caused by the edge effect of top lighting, and affect the nutritional quality of lettuce. B and RGB performed best. There was spatial response diversity of lettuce plants to side lighting spectral qualities.

## 1. Introduction

A plant factory with artificial light (PFAL) is an environmentally controlled facility for horticulture, which can produce high-yield, high-quality, and pesticide-free plants with better water and nutrients efficiency and less labor in small spaces [1]. Controlled environmental conditions and inputs could create optimal conditions for year-round production [2]. In many Asian, European, and North American countries, PFAL is used for the commercial production of leafy vegetables, medicinal herbs, and seedlings [3]. However, the technology of PFAL is still under development, and there are still many areas that need improvement. At present, PFAL still faces many issues, including high initial investment and power costs, low productivity, unsatisfactory flavor and nutritional composition, and so on [4].

Due to the scarcity of spatial resources, PFAL generally adopts a multi-layer cultivation method. Usually, PFAL plants low crops such as leafy vegetables, using top lighting to achieve a spatial distribution of light from top to bottom. This top lighting method has advantages such as high biological photosynthetic efficiency and convenient installation and operation. However, short crops such as leafy vegetables have a high planting density, and lettuce plants compress against each other in the later stages of growth, resulting in dense leaves. By using top lighting, the LED light from the upper part of the canopy cannot penetrate the entire lettuce canopy. In the later stage of plant growth, there is insufficient canopy light, uneven vertical spatial light in the canopy, and particularly insufficient side lighting at the edge of the planting board. This accelerates the aging of the bottom and outer leaves of the canopy, increases the labor cost caused by leaf sorting, reduces the overall photosynthetic efficiency of the plant, and leads to a decrease in yield [5,6,7,8,9]. At the same time, the utilization rate of light is not high, leading to an increase in energy consumption. The top lighting method affects the sale price due to the inconsistent size of lettuce plants caused by the edge effect of PPFD, which is large in the middle and small on both sides of the hydroponic cultivation beds’ planting board.

The top lighting in PFAL is unidirectional lighting and has some disadvantages. Therefore, some supplemental multi-directional and three-dimensional lighting methods have been studied and adopted. On the basis of top lighting, a combination of upward lighting, sideward lighting, and inter-lighting was implemented at the bottom, sides, and inside of the plant canopy to provide different angles of lighting intensity and spectral quality. This could improve the light interception area and utilization efficiency of the plant canopy, optimize the photosynthesis of leaves in different directions, promote plant growth, and improve plant biomass and product nutritional quality [10,11,12,13,14,15,16]. The combination of top lighting and vegetable lower canopy upward lighting could effectively retard senescence and reduce waste, improving the marketable shoot biomass effectively, with red being most efficiently absorbed by chlorophyll. Upward light supplementation could also improve the quality of the leaves, with white, blue, and red, but not green, significantly increasing the ascorbic acid content in the external leaves [6,8]. A similar effect was noted for hemp and roses: upward supplementation of red and blue LED lamps at the bottom of the canopy on hemp plant flower buds significantly increased the content of functional substances and yield in the lower flower bud tissue, and on roses, significantly promoted the flowering of roses and maintained leaf photosynthetic capacity [17,18]. However, the irradiated objects of upward lighting at the bottom of the plant are the mature outer leaves of the plant. At the same time, it is difficult for light to penetrate into the grown inner leaves, resulting in low photosynthesis and photosynthetic products of the plant. Moreover, the lighting fixtures for upward lighting, which need to be installed on the surface of the hydroponic cultivation beds’ planting board, are prone to contact with water, posing certain safety hazards and requiring higher ingress protection (IP) measurement, which means high investment costs [5,6,8].

The combination of top lighting and side lighting for plants can significantly improve the vertical spatial lighting intensity and uniformity of the plant canopy, increase the light interception area and utilization efficiency of plants, improve photosynthesis, increase plant biomass and product nutritional quality, enhance the consistency of plant growth morphology, and effectively improve the productivity in plant factories. Side lighting not only enhanced the photosynthesis of plants such as strawberries and chrysanthemum, and deeply promoted their morphological physiology, but also promoted the formation of leaves, branching formation, and earlier flowering period [19,20,21]. However, there have been no reports on the differences in the spatial response of plants to side lighting with different spectral LEDs. Light spectral quality is an important light attribute that affects plant growth, development, yield, and nutritional quality [22,23]. Studying the impact of side lighting with different spectral qualities and spatial distribution on plant growth, development, yield, and nutritional quality is of great significance for establishing efficient three-dimensional lighting methods for plants, and there is no relevant study yet. These experiments were conducted in PFAL, using hydroponic lettuce as the test material to study the effect of side lighting on lettuce growth, yield, and nutritional quality before harvesting. The focus was on clarifying the spatial response diversity of lettuce plants to side lighting, which contributes to the research on the impact of three-dimensional lighting on plant growth and development.

## 2. Materials and Methods

### 2.1. Plant Materials and Growth Conditions

**Figure 1:**
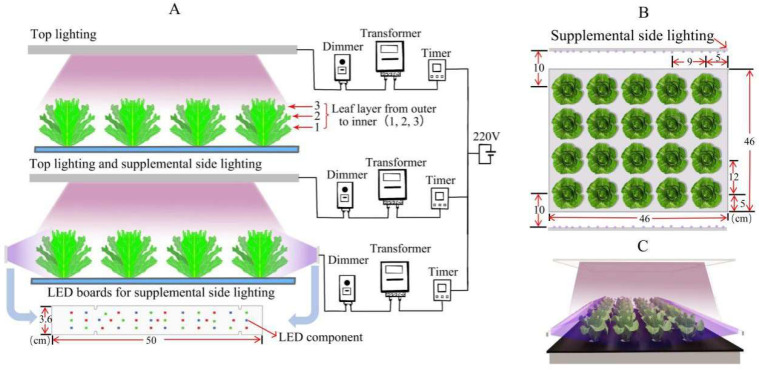
Schematic diagram of the lighting system used in the experiments. There were five groups of cultivation beds with five different experimental lighting treatments, which had two lighting patterns, as top lighting with LED panel and side lighting with line lamps. Each LED panel and line lamp was composed of a digital timer, dimmer, and transformer, used to maintain the lighting period and lighting intensity (**A**). Before the side lighting treatments, the fully expanded third leaves of lettuce plants, grown in the first row and second row at the edge of the top lighting illuminated area, were adjusted toward the side lamps. Starting from the fully expanded third leaves, in a clockwise direction, every three leaves were considered as one layer (just about one circle), and a total of three layers from the outer to the inner were collected, named layer 1 (eldest leaves), layer 2 (middle leaves), and layer 3 (young leaves) (**A**). The control (CK) group remained only top lighting. Four groups were exposed to both top lighting as CK and side lighting (**A**,**C**). The side lighting was employed for an additional 2 h per day after normal top lighting for 6 days before harvest. Each cultivation bed had four rows, and each row had five lettuce plants. The layout of lettuce plants and the relative parameters are shown in (**B**). Line lamps were installed 10 cm from the first row of hydroponic pots at the center of the cultivation panel on the side (**B**).

The experiments were conducted from 6 June to 21 July 2023 in the PFAL of the Institute of Environment and Sustainable Development in Agriculture, Chinese Academy of Agricultural Sciences. Temperature, average relative humidity, and CO_2_ were maintained at 22 ± 1 °C, 50–60%, and 421–425 μmol^−1^, respectively. Seeds of lettuce (*Lactuca sativa* L. cv. “Yidali”) were seeded on sponge cubes (2.5 cm^3^) soaked in Hoagland nutrition solution in plastic germination trays and incubated in darkness for 2 days. The seedlings germinated for 15 days under photosynthetic photon flux density (PPFD) of 200 μmol·m^−2^·s^−1^, provided by the top illuminated white LED lamp board, with a photoperiod of 16 h (6:00–22:00), and the hanging height was 45 cm above the cultivation plant plate. The size of the LED lamp board was 50 × 50 cm^2^ (Zhongshan Zhongfu Plant Technology Co., Ltd., Zhongshan, China) [24]. White LEDs were composed of 15.8% blue (400–500 nm), 22.0% green (500–600 nm), 50.4% red (600–700 nm), and 11.8% far red (700–800 nm). After the second leaf had fully expanded, the seedlings were transplanted from the germination tray to a circulating hydroponic system for 15 days. The system consisted of five sets of hydroponic cultivation beds, a nutrient circulation system, top lighting LED panel, and automatic control lighting system. The seedlings were grown in a deep-flow hydroponic circulation system in Hoagland formula nutrient solution with an electrical conductivity of 1.8 ± 0.1 dS m^−1^ and a pH of 6.8 ± 0.2. Each group had four rows, with five lettuce plants in each row, as shown in Figure 1. Under a 16 h photoperiod (0:00–16:00), the average PPFD of top lighting was 200 μmol·m^−2^·s^−1^, and the ratio of red to blue light intensity was 4R:1B, which was suitable for the growth of lettuce according to previous articles [25]. The top lighting LED panel provided red light with a peak wavelength of 660 ± 2 nm and blue light with a peak wavelength of 445 ± 2 nm. The size of the LED panel was 50 × 50 cm^2^ (Wuxi Huazhaohong Optoelectronics Technology Co., Ltd., Wuxi, China). The LED panel was installed 32 cm above the cultivation plant plate. An LI-1500 irradiance measuring instrument (Lincoln, NE, USA) was used to monitor the average PPFD and spectrum of top lighting and side lighting.

### 2.2. Lighting Treatments

**Figure 2:**
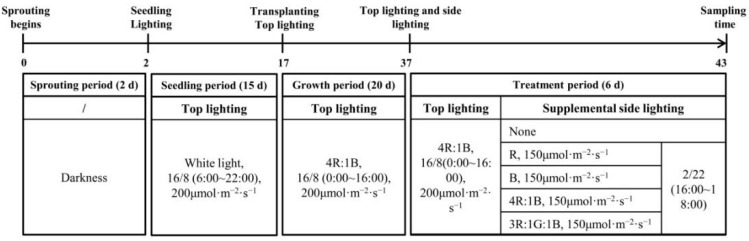
The starting time of sprouting period, seedling period, growth period, treatment period, and sampling time in the experiments.

**Figure 3:**
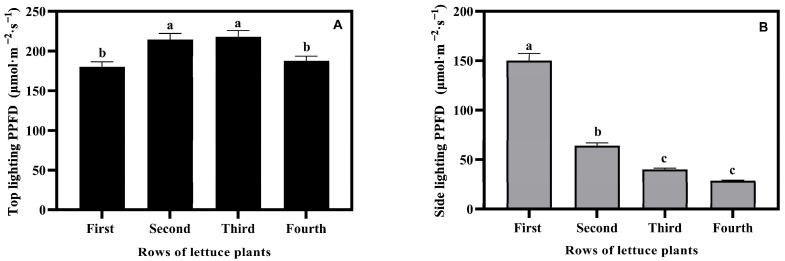
The distribution of PPFD for top lighting (**A**) and side lighting (**B**) of lettuce plants in different rows over the illuminated area of the plant plate of hydroponic cultivation beds. The top lighting PPFD was measured 10 cm above the center of the water culture pot of all rows on the cultivation plant plate, where the orientation of measurement was toward the top light lamps and without any lettuce plants. Side lighting PPFD was measured 10 cm above the center of the water culture pot of first row on the cultivation plant plate, where the orientation of measurement was toward side lamps and without any lettuce plants. Bars represent the standard deviation (n = 5). Different small letters represent significant differences between treatments within different rows of lettuce plants by Duncan’s multiple range test at *p* ≤ 0.05.

After a 20-day cultivation period, the lettuce plants in the five cultivation beds received different experimental lighting treatments. As shown in Figure 1, before the side lighting treatments, the fully expanded third leaves of lettuce plants, grown in the first row and second row at the edge of the top lighting illuminated area (named first row and second row), were adjusted toward the side lamps. Starting from the fully expanded third leaves, in a clockwise direction, every three leaves were considered as one layer (just about one circle), and a total of three layers from the outer to the inner were collected, named layer 1 (eldest leaves), layer 2 (middle leaves), and layer 3 (young leaves). The control (CK) group only maintained top lighting, with an average PPFD of 200 μmol·m^−2^·s^−1^ within a 16 h photoperiod (0:00–16:00), the ratio of red to blue light intensity was 4R:1B. Four groups were exposed to both top lighting and side lighting. As shown in Figure 2, the side lighting of different quality line lamps’ PPFD was 150 μmol·m^−2^·s^−1^, with a photoperiod of 2 h (16:00–18:00). The side lighting line lamps adopted a combination of red, green, and blue LED components (Foshan Haolaite Optoelectronics Technology Co. Ltd., Foshan, China). The size of the side lighting line lamps was 50 × 3.6 × 2.4 cm^3^, with a red-light wavelength of 660 ± 2 nm and a green-light wavelength of 525 ± 2 nm. The wavelength of blue light was 445 ± 2 nm. At the end of irradiation, samples were taken from the leaf layers 1, 2, and 3 of each plant with eliminated petiole. Each leaf layer had three leaves and was mixed as biological replicates. The collected leaves were immediately frozen in liquid nitrogen and stored at −80 °C for future analysis. There were five different experimental lighting treatments, represented as CK, R, B, RB, and RGB (Figure 2). CK denoted lettuce plants grown only under top lighting of 200 μmol·m^−2^·s^−1^ PPFD (4R:1B, PPFD), without any side lighting treatment (Figure 3A), while R, B, RB, and RGB denoted plants grown under both top lighting as CK and with side lighting of red, blue, red:blue (4R:1B, PPFD), and red:green:blue (3R:1G:1G, PPFD) at 150 μmol·m^−2^·s^−1^ PPFD of the first row lettuce and at 60 μmol·m^−2^·s^−1^ PPFD of the second row lettuce (Figure 3B). The side lighting was employed for an additional 2 h per day after normal top lighting for 6 days before harvest. The top lighting PPFD was measured 10 cm above the center of the water culture pot of all rows on the cultivation plant plate, where the orientation of measurement was toward the top light lamps and without any lettuce plants. Side lighting PPFD was measured 10 cm above the center of the water culture pot of first row on the cultivation plant plate, where the orientation of measurement was toward side lamps and without any lettuce plants.

### 2.3. Yield, Leafy Morphology, and Chlorophyll Measurements

At 43 days after sowing, lettuce plants were harvested, and four lettuce plants in the first row and second row toward the side lamps were sampled under five different experimental lighting treatments. Firstly, the chlorophyll content was evaluated using SPAD-502 (Tokyo, Japan) at the same leaf position. Then, shoot fresh weigh of whole plant and fresh weight of leaf layers, named layer 1, 2 and 3, were determined using an electronic balance (precision: 0.001 g). Leaf area of leaf layer 1, 2, and 3 and whole plant were measured by LI-3000 C leaf area meter (LI-COR). Lastly, the blades (without petioles) were frozen with liquid nitrogen rapidly, and ground into powder with a high-throughput tissue grinder (SCIENTZ-48, Ningbo, China) at low temperature. The powdered samples were stored in a refrigerator at −80 °C for future use.

### 2.4. Nitrate Measurement

The nitrate content was measured using the improved method proposed by Cataldo et al. [26]. We added 1.5 mL of deionized water to 0.1 g of the powered sample, extracted in a boiling water bath at 100 °C for 30 min, and subsequently cooled it down. Then, we used a centrifuge (Sigma 3K30, Burladingen, Germany) to centrifuge the sample (13,000 rpm, 15 min) to separate the supernatant. Then, 0.1 mL of the supernatant with 0.4 mL of 5% (*w*/*v*) salicylic acid (in pure H_2_SO_4_) and 9.5 mL of 8% NaOH were mixed. Finally, we used a microplate reader (Infinite 200 PRO, TECAN, Mannedorf, Switzerland) to measure the absorbance extracted at 410 nm to determine the nitrate content. The nitrate content was calculated on the basis of the measured absorbance and the nitrate calibration curve. The measurement unit of nitrate content was mg/g of fresh weight.

### 2.5. Soluble Sugar and Starch Measurements

The soluble sugar content was determined using the Anthrone colorimetric method [27]. Initially, 0.1 g of the powered sample was added to 1.5 mL of deionized water, and then extracted in water at 100 °C for 30 min before cooling. We vortexed the homogenate thoroughly and centrifuged it using a centrifuge at 15,000 rpm for 15 min. We combined 0.4 mL of the supernatant with 1.6 mL of deionized water and 0.5 mL of Anthrone ethyl acetate solution. We added 5 mL of concentrated sulfuric acid, shook the reaction mixture vigorously, and then soaked it in boiling water for 1 min. Finally, a microplate reader was used to measure the extraction absorbance at 630 nm to determine the soluble sugar content. The soluble sugar content was calculated on the basis of the measured absorbance and the soluble sugar calibration curve. The measurement unit of soluble sugar content was mg/g of fresh weight.

Quantitative analysis of starch was determined using a starch content determination kit (Beijing Solarbio Science & Technology Co., Ltd., Beijing, China). We added 1 mL of 80% ethanol to a total of 0.1 g of powered sample. The samples were extracted in a water bath at 80 °C for 30 min to separate soluble sugars and starch from the samples. After centrifuging with a centrifuge (15,000 rpm, room temperature, 10 min), we discarded the supernatant and retained the sediment. Then, the starch was decomposed into glucose through acid hydrolysis, and the starch content was determined using the Anthrone colorimetric method. The starch content was calculated on the basis of the measured absorbance and the starch calibration curve. The measurement unit of starch content was mg/g of fresh weight.

### 2.6. Flavonoid and Phenolics Measurements

We measured the total flavonoid content according to the method described by Shin et al. [28]. We weighed 0.1 g of powder sample and 1.5 mL of 80% methanol and added them to a 2 mL centrifuge tube. We performed ultrasonic extraction in a dark environment for 20 min using an ultrasonic device. We thoroughly vortexed the homogenate and centrifuged it using a centrifuge (15,000 rpm, 4 °C, 5 min). We mixed 0.1 mL of supernatant with 0.4 mL of water, then added 30 μL of 5% NaNO_2_ solution. Subsequently, after 5 min, we added 30 μL aluminum chloride and 200 μL 1 mol/L NaOH at the same time. Finally, we used a microplate reader to measure the absorbance extracted at 350 nm to determine the content of flavonoid substance. The total flavonoid content was calculated on the basis of the measured absorbance and the total flavonoid calibration curve. The measurement unit of total flavonoid content was mg/g of fresh weight.

The total phenolics content was determined using the Folin–Ciocalteu method. We weighed 0.1 g of powered sample and 1.5 mL of 80% methanol and added them to a 2 mL centrifuge tube. We performed ultrasonic extraction in a dark environment for 20 min using an ultrasonic device. We thoroughly vortexed the homogenate and centrifuged it using a centrifuge (15,000 rpm, 4 °C, 5 min). We mixed 0.1 mL of supernatant with 0.8 mL of 0.1 mol/L Folin–Ciocalteu reagent. After vigorous shaking, we let the mixture stand for 5 min. Subsequently, we added 0.8 mL of 7% NaCO_3_ solution and left the mixture at room temperature in the dark for 90 min until it cooled naturally to room temperature. Finally, we used a microplate reader to measure the absorbance extracted at 750 nm to determine the content of phenolic substance. The total phenolics content was calculated on the basis of the measured absorbance and the total phenolics calibration curve. The measurement unit of total phenolics content was mg/g of fresh weight.

### 2.7. Statistical Analysis

The data was analyzed using SPSS software version 22.0 (IBM Corporation, New York, NY, USA). We performed one-way analysis of variance (ANOVA) on the data and used the Duncan multiple range test at a 95% confidence level to determine significant differences among treatments.

## 3. Results

### 3.1. Growth, Yield, Chlorophyll Content, and Nitrate Content

**Figure 4:**
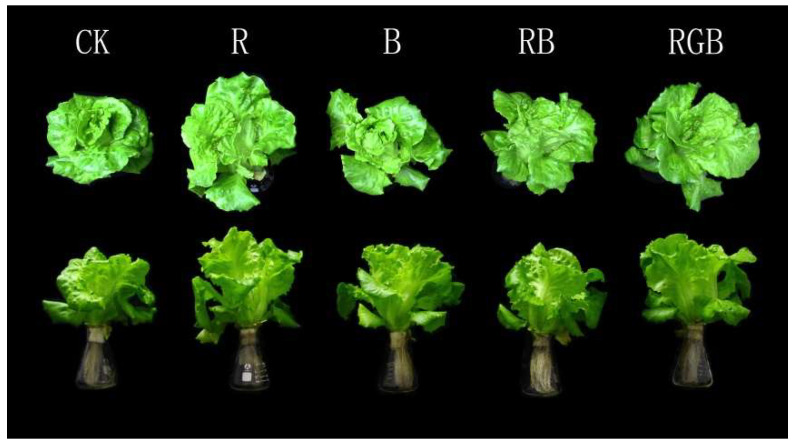
Growth status of lettuces in the first row under top lighting, with or without side lighting of different qualities at 150 μmol·m^−2^·s^−1^ PPFD after 6 days’ lighting treatment, measured 10 cm above the center of the water culture pot of the first row toward the side lamps.

In this assay, 37-day-old seedlings were employed under different types of supplemental side lighting. It was obvious that under CK treatment, the growth morphology of lettuce canopy in the first row was compact, while under side lighting, the lettuce canopy was relatively loose and had a large crown width, and the outer leaves of lettuce plants grew toward the side lamps (Figure 4).

**Figure 5:**
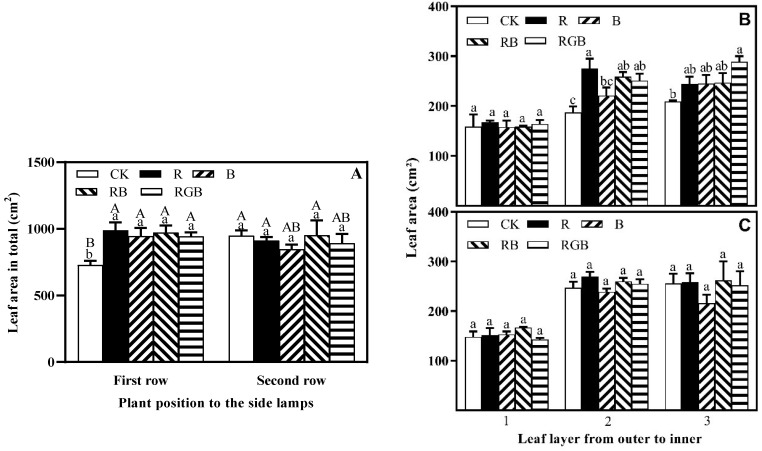
Leaf area in total plants (**A**), leaf area of the leaf layers (from outer to inner) of plants, grown in the first (**B**) and second (**C**) row toward side lamps, respectively, with or without supplemental side lighting of different qualities at 150 μmol·m^−2^·s^−1^ PPFD, measured 10 cm above the center of the water culture pot of the first row toward the side lamps. Bars represent the standard deviation (n = 4). Different capital letters represent significant differences between treatments and rows by Duncan’s multiple range test at *p* ≤ 0.05 (**A**). Different small letters represent significant differences between treatments within each leaf layer separately by Duncan’s multiple range test at *p* ≤ 0.05.

R, B, RB, and RGB treatments significantly increased the leaf area of the first row of lettuce plants by 36%, 30%, 33%, and 30% compared to the CK, respectively (Figure 5A). However, there was no significant difference between R, B, RB, and RGB treatments, and there was no significant difference in the leaf area of the second row of lettuce plants under different treatments. R, B, RB, and RGB treatments improved the leaf area in total of lettuce plants grown at the first and second row (Figure 5A). 

There was no significant difference in the leaf area of leaf layer 1 of the first row of lettuce plants, closest to the side lamps, while leaf layer 2 and leaf layer 3 of lettuce increased significantly (Figure 5B,C). There was no significant difference in the leaf area of leaf layers 1, 2, and 3 of the second row of lettuce plants. In leaf layer 2 of the first row of lettuce plants, the leaf area under R, RB, and RGB treatments significantly increased by 47%, 39%, and 34%, respectively, with R treatment increasing the most. In leaf layer 3 of the first row of lettuce plants, the leaf area under RGB treatments increased the most, by 47%. Overall, compared to CK treatment, the R, B, RB, and RGB treatments affected the leaf area of leaf layer 2 of the first row the most, followed by leaf layer 3, and leaf layer 1 less.

**Figure 6:**
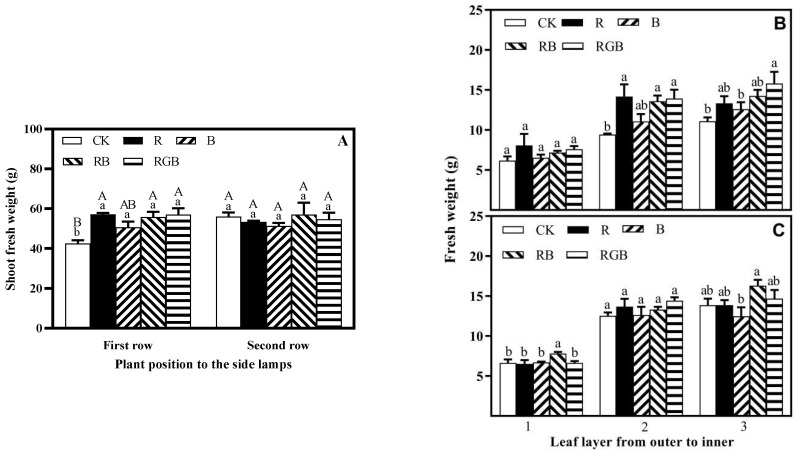
Shoot fresh weigh of whole plant (**A**) and fresh weight of leaf layer, grown in the first (**B**) and second (**C**) row toward side lamps, respectively, with or without supplemental side lighting of different qualities at 150 μmol·m^−2^·s^−1^ PPFD, measured 10 cm above the center of the water culture pot of the first row toward the side lamps. Bars represent the standard deviation (n = 4). Different capital letters represent significant differences between treatments and rows by Duncan’s multiple range test at *p* ≤ 0.05 (**A**). Different small letters represent significant differences between treatments within each leaf layer separately by Duncan’s multiple range test at *p* ≤ 0.05.

Compared with CK treatment, with supplemental side lighting, R, B, RB, and RGB resulted in a significant increase of the shoot fresh weight in the first row of lettuce plants by 34%, 19%, 31%, and 34%, respectively (Figure 6A), and there was no significant difference in fresh weight among R, B, RB, and RGB treatments. However, there was no significant improvement in the shoot fresh weight of the second row of lettuce plants under different treatments. R, B, RB, and RGB treatments improved the shoot fresh weight consistency of lettuce plants grown at the first and second row (Figure 6A). R, RB, and RGB treatments significantly increased the fresh weight of leaf layer 2 and leaf layer 3 of the first row of lettuce plants, which increased by 50%, 44%, and 48% in the leaf layer 2 leaves, and increased by 20%, 29%, and 42% in the leaf layer 3 leaves, in comparison with CK treatment, respectively (Figure 6B,C). The fresh weight of leaf layer 1 of the first row of lettuce plants’ increase was not significant. B treatments did not significantly increase the fresh weight of leaf layer 3 of the first row of lettuce plants. Under the RB treatment, the fresh weight of leaf layer 1 and leaf layer 3 of the second row of lettuce plants significantly increased, while the effects of other treatments were not significant. Comprehensively, R, B, RB, and RGB treatments affected the fresh weight of leaf layer 2 of the first row the most, followed by leaf layer 3, and leaf layer 1 less.

**Figure 7:**
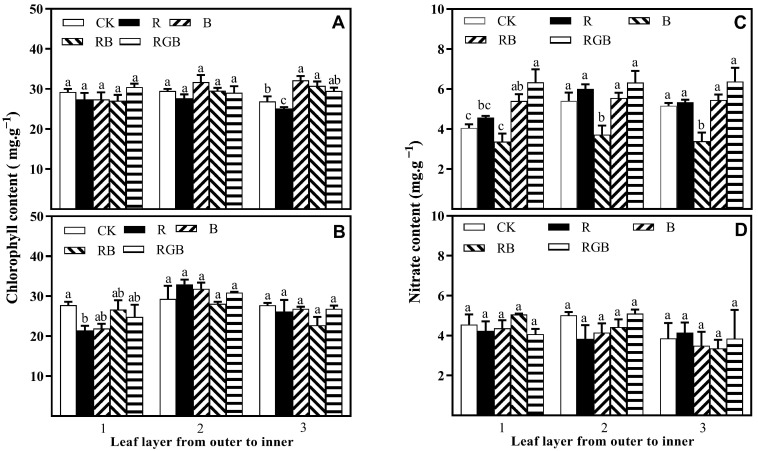
Chlorophyll content and nitrate content in the leaf layers (from outer to inner) of plants, grown in the first (**A**,**C**) and second (**B**,**D**) row toward side lamps, respectively, with or without side lighting of different qualities at 150 μmol·m^−2^·s^−1^ PPFD, measured 10 cm above the center of the water culture pot of the first row toward the side lamps. Bars represent the standard deviation (n = 4). Different small letters represent significant differences between treatments within each leaf layer separately by Duncan’s multiple range test at *p* ≤ 0.05.

There was no significant difference in chlorophyll content between leaf layer 1 and leaf layer 2 of the first row of lettuce plants, while there was a significant difference in the chlorophyll content of leaf layer 3: among them, B, RB, and RGB treatments significantly increased leaf chlorophyll content, while R treatment had the opposite effect (Figure 7A,B). The difference in the chlorophyll content of leaf layer 2 and leaf layer 3 of the second row of lettuce plants was not significant (Figure 7A,B). The chlorophyll content in leaf layer 1 of the second row of lettuce plants decreased, and the reduction was highest under R treatment.

Supplemental side lighting of RB and RGB significantly increased the nitrate content in leaf layer 1 of the first row of lettuce plants, while B significantly reduced the nitrate content in the leaf layer 2 and leaf layer 3 (Figure 7C,D). No significant difference among treatments was observed in the nitrate content of the second row of lettuce plants.

### 3.2. Sugar and Starch Contents

**Figure 8:**
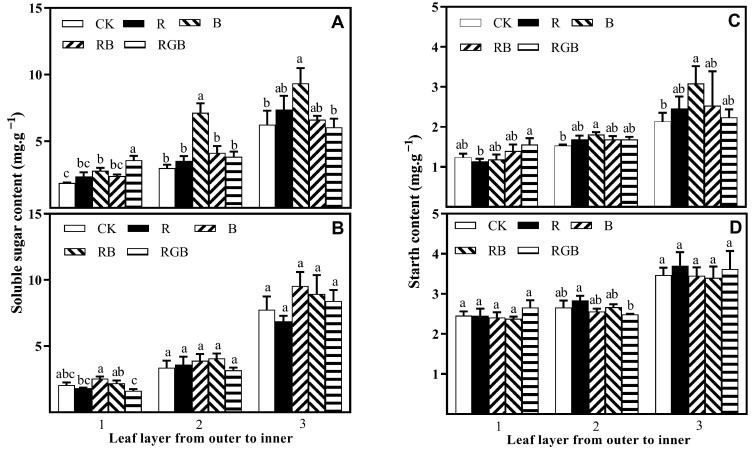
Soluble sugar content and starch content in the leaf layers (from outer to inner) of plants, grown in the first (**A**,**C**) and second (**B**,**D**) row toward side lamps, respectively, with or without side lighting of different qualities at 150 μmol·m^−2^·s^−1^ PPFD, measured 10 cm above the center of the water culture pot of the first row toward side lamps. Bars represent the standard deviation (n = 4). Different small letters represent significant differences between treatments within each leaf layer separately by Duncan’s multiple range test at *p* ≤ 0.05.

Supplemental side lighting resulted in significantly higher soluble sugar content in leaf layer 1 of the first row of lettuce plants. B treatment significantly increased the soluble sugar content of leaf layers 1, 2, and 3 of the first row of lettuce plants, as well as leaf layer 1 of the second row of lettuce plants (Figure 8A,B). Under RGB treatment, the soluble sugar content in leaf layer 1 of the first row of lettuce plants was increased by 91%, but leaf layer 1 of the second row of lettuce plants showed the opposite effect (Figure 8A,B). There was no significant difference in the soluble sugar content of leaf layer 2 and leaf layer 3 of the second row of lettuce plants under different treatments.

Supplemental side lighting, besides R, resulted in significantly higher starch content in leaf layers 1, 2, and 3 of the first row of lettuce plants. Compared to CK, B treatment significantly increased the starch content of leaf layer 2 and leaf layer 3 of the first row of lettuce plants, and leaf layer 3 showed the highest increase, at 45% (Figure 8C,D). RGB treatment significantly increased the starch content of leaf layer 1 of the first row of lettuce plants, but significantly decreased the starch content of leaf layer 2 of the second row of lettuce plants, while R treatment had the opposite effect completely (Figure 8C,D). No significant difference was observed in the starch content of leaf layer 1 and leaf layer 3 of the second row of lettuce plants under different treatments.

### 3.3. Flavonoid and Phenolics Contents

**Figure 9:**
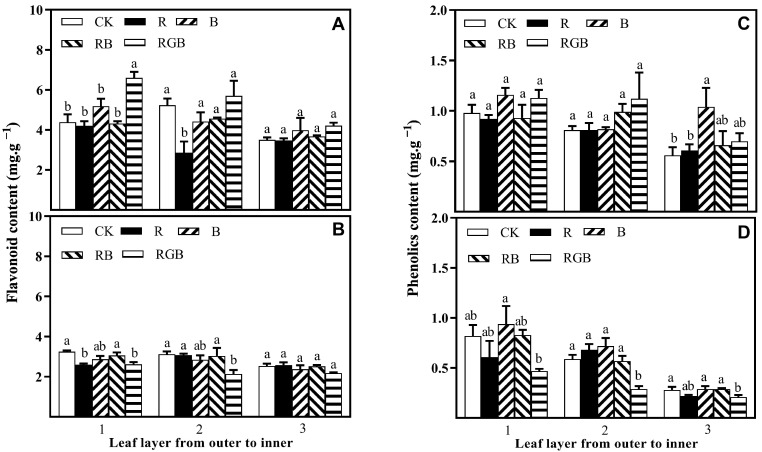
Flavonoid content and phenolics content in the leaf layers (from outer to inner) of plants, grown in the first (**A**,**C**) and second (**B**,**D**) row toward side lamps, respectively, with or without side lighting of different qualities at 150 μmol·m^−2^·s^−1^ PPFD, measured 10 cm above the center of the water culture pot of the first row toward side lamps. Bars represent the standard deviation (n = 4). Different small letters represent significant differences between treatments within each leaf layer separately by Duncan’s multiple range test at *p* ≤ 0.05.

The flavonoid content in leaf layer 1 of the first row of lettuce plants significantly increased by 50% with RGB treatment, and decreased in leaf layer 1 and 2 of the second row of lettuce plants (Figure 9A,B), compared with CK. R treatment significantly reduced the flavonoid content in leaf layer 2 of the first row of lettuce plants and leaf layer 2 of the second row of lettuce plants. There was no significant difference in the flavonoid content of the lettuce plants under B and RB treatments.

Supplemental side lighting of B significantly increased the total content of phenolic compounds the leaf layer 3 of the first row of lettuce plants and leaf layer 1 of the second row of lettuce plants, with layer 3 increasing by 87% in comparison with CK (Figure 9C,D). RGB treatment significantly depressed the total content of phenolic compounds in leaf layers 1, 2, and 3 of the second row of lettuce plants.

## 4. Discussion

### 4.1. Side Lighting Increased Lettuce Yield Irrespective of Spectral Qualities 

Using only top lighting, the LED lights on the plant plate of the hydroponic cultivation beds cannot effectively penetrate the entire plant canopy, resulting in a lack of sufficient upward and sideward lighting intensity at the bottom and outside of the plant canopy, accelerating leaf aging at both the bottom and side leaves of the plant canopy, reducing the overall photosynthetic efficiency of the plant, and reducing, with a loss of up to 10%, plant yield. It also increases the power cost of plant lighting and the labor cost of pruning aging leaves [5,6,29]. Studying the positive effects of side lighting on maintaining production stability and achieving high yield in PFAL, especially exploring the relationship between the yield increase benefits and energy consumption of short-term side lighting, has practical production value.

Our results clearly indicated that side lighting with different spectral LEDs retarded the outer leaf senescence of lettuce and improved the growth and yield of the lettuce plants grown in the first row toward the side lamps (Figure 6A). In relevant studies, it was also found that side lighting had a significant impact on plants. Seedlings of two head lettuce (*Lactuca sativa* L.) cultivars grown under side lamps of white light achieved the largest shoot fresh weight, compared with top lighting and upward lighting [19]. Under the condition of supplementing with an adjustable sideward lighting system of white light, the number of leaves, stem diameter, fresh dry weight, chlorophyll a and b concentrations, and biochemical content (soluble sugars and proteins) of romaine lettuce all increased sharply [30]. Photosynthesis is one of the most important chemical reactions in plants, and PSI and PSII are two systems driven by light energy, playing a synergistic role in primary energy conversion reactions [31,32,33,34,35]. In this study, supplementing short-term side lighting before lettuce harvesting could significantly improve the lighting intensity of the lettuce plants in the first row toward the side lamps, and possibly increase lettuce photosynthesis, thereby increasing yield.

Lighting is one of the main energy inputs in PFAL systems that needs to be optimized to reduce costs [35]. One advantage of LED lighting used in vegetable production in PFAL systems is the ability to synthesize spectra crucial for plant growth while minimizing unnecessary spectra [36]. In order to evaluate the economic feasibility of planting lettuce in a PFAL system with different qualities of side lighting, the ratio of light energy consumption to fresh weight increase was studied. Compared with the CK group, after short-term side lighting with different spectral qualities of 150 μmol·m^−2^·s^−1^, for 2 h per day after normal top lighting for 6 days before harvest, the fresh weight of five lettuce plants in the first row increased by 73.35 g, 40.85 g, 67.00 g, and 72.35 g, under R, B, RB, and RGB lighting treatments, respectively. The ratio of light energy consumption to fresh weight increase under R, B, RB, and RGB lighting treatments was 1.61, 3.55, 2.00, and 2.13 kW·h·kg^−1^, respectively, which was much lower than that of only the traditional top lighting of 8–10 kW·h·kg^−1^ [29]. Therefore, short-term side lighting can significantly increase lettuce yield, increase the ratio between the production output and input of light energy consumption, and has good practical application value. 

### 4.2. Side Lighting Affected Lettuce Nutritional Quality, Varying with Spectral Qualities 

Nitrate is an important component of vegetables because of its potential for accumulation. Higher levels of nitrate tend to be found in leaves, whereas lower levels occur in seeds or tubers. Therefore, leaf crops such as lettuce and spinach typically have higher nitrate concentrations [37,38,39,40]. In the complex process of carbon and nitrogen metabolism in plants, the lighting environment plays a decisive role. High soluble sugar content might be the key to supporting sustained nitrogen metabolism, thereby reducing nitrate accumulation [41,42,43,44,45,46]. The lighting environment conditions, such as lighting spectral quality composition, mainly regulate the metabolism and distribution of carbon and nitrogen in plants by affecting their photosynthesis and leaf morphogenesis. Red light could enhance the activity of nitrate reductase in plant leaves, accelerate nitrate metabolism, and promote the conversion of inorganic nitrogen to organic nitrogen [47]. Blue light could increase nitrate reductase (NR) activity, invertase activity and protein, and strengthen N metabolism [48]. Compared with monochromatic red light treatment, the combination of red and blue light has been found to be more conducive to reducing the accumulation of nitrate in lettuce and increasing the content of soluble sugar [49,50,51,52]. In this study, the side lighting spectrum might have similar impacts on plants as those of the top lighting spectrum, due to the approximate lighting intensity. For leaf layers 2 and 3 in the first row of lettuce plants, the highest levels of soluble sugar content might possibly help increase nitrate reductase content and promote nitrate decomposition [44,48], when supplemented with side blue light, and the decomposition effect of nitrate might reduce the content of nitrate.

Phenols, including flavonoids and anthocyanins, are powerful antioxidants with the ability to resist DNA oxidative damage and prevent human chronic diseases. The number and location of hydroxyl groups in the flavonoid structure seem to be important for the antioxidant and cellular protective potential of compounds [53]. Compared to white, green, and red light, blue light irradiation was crucial for the accumulation of phenolic compounds in secondary barley leaves [54]. The expression levels of dihydroflavonol 4-reductase and anthocyanin synthase genes were higher in young leaves, but gradually decreased with leaf development, and were lowest in mature leave [55]. In our study, a similar result showed that supplementation of side blue light significantly increased the phenolic content of leaf layer 3 (young leaves) in the first row of lettuce plants. 

Supplement of side RGB light significantly increased the flavonoid content of leaf layer 1 in the first row of lettuce plants, while supplement of side red light significantly reduced the flavonoid content in leaf layer 2 of the first row of lettuce plants. R and RGB light did not significantly affect the flavonoid and phenolic content in other leaf layers of the first row of lettuce plants. Similar results have also been reported in some relevant reports. The accumulation rate of total flavonoids of *S. vaninii* under blue light was faster, while the total flavonoid accumulation rate under red light was slower than under white light, and yellow and green lights had little effect on the synthesis of total flavonoids [56]. Dou H. et al. [57] found that the concentration of anthocyanins, phenols, and flavonoids in green basil leaves, as well as antioxidant capacity, did not differ between treatments of R_44_B_24_G_32_, but decreased under treatments of R_74_B_16_G_10_ and R_42_B_13_G_45_. It was recommended to use white light with a low green light ratio (10%) for basil production. Our experimental results were consistent with these studies. A certain amount of G light was beneficial for promoting the growth of lettuce, but it could not significantly increase the content of flavonoids and total phenols in lettuce, while red light reduced the content of flavonoids and total phenols in lettuce.

### 4.3. Spatial Response Diversity of Lettuce Plants to Different Side Lighting Spectral Qualities

The spatial distribution of light has a complex impact on the photosynthesis, nutritional quality, and yield of plants [15]. This emphasizes the importance of optimizing the spatial distribution of light in plant cultivation. This is particularly important for plants growing under artificial lighting conditions, such as lettuce, where the design of light spatial distribution is crucial [58]. In this study, compared to CK, side lighting significantly increased the shoot fresh weight and leaf area of the first row of lettuce plants irrespective of spectral quality, while the increase in the second row of lettuce plants was not significant. This indicates that there was a spatial diversity of responses of shoot fresh weight and leaf area in different lettuce plant rows to side lighting, which was independent of spectral quality. Through side lighting, the fresh weight and leaf area of the first and second rows of lettuce were similar, effectively improving the inconsistent size of lettuce plants caused by the edge effect of top lighting. In addition, due to the obstruction of the lettuce in the first row, the partial side light could only transmit and reflect to the second row of lettuce plants from the planting box wall and planting board; therefore, the promotion of fresh weight and leaf area of lettuce plants in the second row was not significant. Our research results confirmed that the combination of top lighting and side lighting significantly increased the side lighting intensity of the lettuce canopy in the first row toward the side lamps, creating horizontal and vertical lighting for lettuce plants grown on the hydroponic cultivation beds’ planting board.

Side lighting increased the yield of the lettuce grown in the first row irrespective of spectral qualities. However, the yield increase of different leaf layers of lettuce grown in the first row was related to the type of spectral qualities. Supplemental side R light had the best effect on improving the fresh weight of leaf layer 2 of lettuce of the first row, followed by RGB and RB. All spectral qualities had no significant effect on leaf layer 1 of the first row. This was the same trend as that found in the results of Shimizu, H. and Lee et al. [59,60], which provide a basis for the results of the longest leaf length and the largest number of leaves found in lettuce grown under red light alone. Supplemental upward lighting of red retarded senescence and reduced waste, improving the marketable shoot biomass effectively, as red is most efficiently absorbed by chlorophyll [7,8]. Compared to other spectra, supplemental side RGB light increased the fresh weight of leaf layer 3 of the first row most. Under high PPFD, compared to red and blue light, green light was evenly distributed throughout the entire spectrum (red, green, and blue) into the plant canopy and lower leaves, exhibiting a higher CO_2_ assimilation rate. It has been reported that lettuce plants produced the highest fresh biomass when 30 μmol·m^−2^·s^−1^ PPFD of G light was supplemented with R and B light, maintaining the ratio 211:30:53 (R:G:B); it was suggested that an LED combination of 72% red, 10% green, and 18% blue was suitable for growing lettuce under artificial light [61,62]. In lettuce plants, a combination of red and blue LED light with green light was found to be more productive (about 20%) than RB light alone [31]. In this study, supplemental side RGB light increased the fresh weight of leaf layer 3 of the first row of lettuce the most, and appropriate G light might help improve the photosynthesis of lettuce. 

## 5. Conclusions

To summarize, short-term side lighting before lettuce harvest could significantly improve the side lighting intensity, and increase the shoot fresh weight and leaf area of lettuce plants, effectively improving the inconsistent size of lettuce plants caused by the edge effect of top lighting. Besides yield improvement by side lighting, B treatment significantly reduced the nitrate content and increased soluble sugar contents, starch contents, and the content of phenolics in leaf layer 3 of the first row of lettuce plants. RB and RGB significantly increased the flavonoid content of leaf layer 1 of the first row of lettuce plants, while R had opposite effect completely. Supplemental short-term side lighting, irrespective of spectral qualities, could effectively promote yield, especially for leaf layers 2 and 3 of the first row of lettuce plants, and affect the nutritional quality of lettuce. B and RGB performed best. Our results demonstrate that there was spatial response diversity of lettuce plants to side lighting spectral qualities, and short-term side lighting with appropriate spectral quality can be used to promote the growth, yield, and nutritional quality of vegetables in a plant factory.

## Data Availability

Please contact the corresponding author for any additional information.

## References

[1] Brandon M., Kozai T., Lu N., Yamaguchi T., Takagaki M., Maruo T., Yamori W., Pessarakli M. (2016). Next revolution of agriculture: A review of innovations in plant factories. Handbook of Photosynthesis.

[2] Yamori W., Zhang G., Takagaki M., Maruo T. (2014). Feasibility study of rice growth in plant factories. J. Rice Res..

[3] Hayashi E., Kozai T., Fujiwara K., Runkle E.S. (2016). Current status of commercial plant factories with LED lighting market in Asia, Europe, and other regions. LED Lighting for Urban Agriculture.

[4] Kozai T. (2013). Resource use efficiency of closed plant production system with artificial light: Concept, estimation and application to plant factory. Proc. Jpn. Acad. Ser. B..

[5] Zhang G., Shen S., Takagaki M., Kozai T., Yamori W. (2015). Supplemental upward lighting from underneath to obtain higher marketable lettuce (*Lactuca Sativa*) leaf fresh weight by retarding senescence of outer leaves. Front. Plant Sci..

[6] Joshi J., Zhang G., Shen S., Supaibulwatana K., Watanabe C.K.A., Yamori W. (2017). A combination of downward lighting and supplemental upward lighting improves plant growth in a closed plant factory with artificial lighting. HortScience.

[7] Saengtharatip S., Lu N., Takagaki M. (2018). Supplemental upward LED lighting for growing romaine lettuce (*Lactuca Sativa*) in a plant factory: Cost performance by light intensity and different light spectra. Acta Hortic..

[8] Saengtharatip S., Joshi J., Zhang G., Takagaki M., Kozai T., Yamori W. (2021). Optimal light wavelength for a novel cultivation system with a supplemental upward lighting in plant factory with artificial lighting. Environ. Control. Biol..

[9] Saito K., Goto E. (2023). Evaluation of the enhancement of photosynthetic rate in a komatsuna (*Brassica rapa* L. var. *perviridis*) canopy with upward lighting using an optical simulation in a plant factory with artificial light. Front. Plant Sci..

[10] Frantz J.M., Joly R.J., Mitchell C.A. (2000). Intracanopy lighting influences radiation capture, productivity, and leaf senescence in cowpea canopies. J. Am. Soc. Hortic. Sci..

[11] Hovi-Pekkanen T., Tahvonen R. (2008). Effects of interlighting on yield and external fruit quality in year-round cultivated cucumber. Sci. Hortic..

[12] Kim D., Moon T., Kwon S., Hwang I., Son J.E. (2023). Supplemental inter-lighting with additional far-red to red and blue light increases the growth and yield of greenhouse sweet peppers (*Capsicum annuum* L.) in winter. Hortic. Environ. Biotechnol..

[13] Kwon S., Kim D., Moon T., Son J.E. (2023). Evaluation of the light use efficiency and water use efficiency of sweet peppers subjected to supplemental interlighting in greenhouses. Hortic. Environ. Biotechnol..

[14] Pettersen R.I., Torre S., Gislerød H.R. (2010). Effects of intracanopy lighting on photosynthetic characteristics in cucumber. Sci. Hortic..

[15] Schipper R., Van Der Meer M., De Visser P.H.B., Heuvelink E., Marcelis L.F.M. (2023). Consequences of intra-canopy and top LED lighting for uniformity of light distribution in a tomato crop. Front. Plant Sci..

[16] Wenke L. (2021). Definition and application strategy for stereo lighting in plant factory. China Light Light..

[17] Hawley D., Graham T., Stasiak M., Dixon M. (2018). Improving cannabis bud quality and yield with subcanopy lighting. HortScience.

[18] Yamori N., Matsushima Y., Yamori W. (2021). Upward led lighting from the base suppresses senescence of lower leaves and promotes flowering in indoor rose management. HortScience.

[19] Wang M., Wei H., Jeong B.R. (2021). Lighting direction affects leaf morphology, stomatal characteristics, and physiology of head lettuce (*Lactuca sativa* L.). Int. J. Mol. Sci..

[20] Yang J., Song J., Jeong B.R. (2021). Side lighting enhances morphophysiology and runner formation by upregulating photosynthesis in strawberry grown in controlled environment. Agronomy.

[21] Yang J., Song J., Jeong B.R. (2022). Lighting from top and side enhances photosynthesis and plant performance by improving light usage efficiency. Int. J. Mol. Sci..

[22] Naznin M.T., Lefsrud M., Gravel V., Azad M.O.K. (2019). Blue light added with red LEDs enhance growth characteristics, pigments content, and antioxidant capacity in lettuce, spinach, kale, basil, and sweet pepper in a controlled environment. Plants.

[23] Alrajhi A.A., Alsahli A.S., Alhelal I.M., Rihan H.Z., Fuller M.P., Alsadon A.A., Ibrahim A.A. (2023). The effect of LED light spectra on the growth, yield and nutritional value of red and green lettuce (*Lactuca sativa*). Plants.

[24] Zhou C., Shao M., Liu W., Li B., Wang Q., Liu J., Wen Y., Yang Q. (2021). Regulation of ascorbate accumulation and metabolism in lettuce by end-of-production high light irradiation provided by red and blue LEDs. Environ. Exp. Bot..

[25] Liu J., Liu W. (2022). Regulation of accumulation and metabolism circadian rhythms of starch and sucrose in two leaf-color lettuces by red: Blue ratios of led continuous light. Environ. Exp. Bot..

[26] Cataldo D.A., Maroon M., Schrader L.E., Youngs V.L. (1975). Rapid colorimetric determination of nitrate in plant tissue by nitration of salicylic acid. Commun. Soil Sci. Plant Anal..

[27] Li H., Sun H., Li M. (2018). Prediction of soluble sugar content in cabbage by near infrared spectrometer. Spectrosc. Spectr. Anal..

[28] Shin Y.K., Bhandari S.R., Jo J.S., Song J.W., Cho M.C., Yang E.Y., Lee J.G. (2020). Response to salt stress in lettuce: Changes in chlorophyll fluorescence parameters, phytochemical contents, and antioxidant activities. Agronomy.

[29] Kozai T., Zhang G., Kozai T., Fujiwara K., Runkle E.S. (2016). Some aspects of the light environment. LED Lighting for Urban Agriculture.

[30] Arcel M.M., Yousef A.F., Shen Z.H., Nyimbo W.J., Zheng S.H. (2023). Optimizing lettuce yields and quality by incorporating movable downward lighting with a supplemental adjustable sideward lighting system in a plant factory. PeerJ..

[31] Kozai T., Kino S., Jeong B.R., Kinowaki M., Ochiai M., Hayashi M., Mori K. (1992). A sideward lighting system using diffusive optical fibers for production of vigorous micropropagated plantlets. Acta Hortic..

[32] Wei H., Zhao J., Hu J., Jeong B.R. (2019). Effect of supplementary light intensity on quality of grafted tomato seedlings and expression of two photosynthetic genes and proteins. Agronomy.

[33] Khan M.S., Hameed W., Nozoe M., Shiina T. (2007). Disruption of the *psbA* gene by the copy correction mechanism reveals that the expression of plastid-encoded genes is regulated by photosynthesis activity. J. Plant Res..

[34] Leelavathi S., Bhardwaj A., Kumar S., Dass A., Pathak R., Pandey S.S., Tripathy B.C., Padmalatha K.V., Dhandapani G., Kanakachari M. (2011). Genome-wide transcriptome and proteome analyses of tobacco psaA and psbA deletion mutants. Plant Mol. Biol..

[35] Mitchell C., Both A.J., Bourget C., Burr J., Kubota C., Lopez R., Morrow R., Runkle E. (2012). LEDs: The future of greenhouse lighting!. Chron. Hortic..

[36] Morrow R.C. (2008). LED lighting in horticulture. HortScience.

[37] Bian Z., Cheng R.F., Yang Q.C., Wang J., Lu C. (2016). Continuous light from red, blue, and green light-emitting diodes reduces nitrate content and enhances phytochemical concentrations and antioxidant capacity in lettuce. J. Am. Soc. Hortic. Sci..

[38] Wanlai Z., Wenke L., Qichang Y. (2013). Reducing nitrate content in lettuce by pre-harvest continuous light delivered by red and blue light-emitting diodes. J. Plant Nutr..

[39] Yamori W., Nagai T., Makino A. (2011). The rate-limiting step for CO2 assimilation at different temperatures is influenced by the leaf nitrogen content in several C3 crop species. Plant Cell Environ..

[40] Shao M., Liu W., Zhou C., Wang Q., Li B. (2022). Alternation of temporally overlapped red and blue light under continuous irradiation affected yield, antioxidant capacity and nutritional quality of purple-leaf lettuce. Sci. Hortic..

[41] Huner N.P.A., Öquist G., Sarhan F. (1998). Energy balance and acclimation to light and cold. Trends Plant Sci..

[42] Scaife A., Schloemer S. (1994). The diurnal pattern of nitrate uptake and reduction by spinach (*Spinacia oleracea* L.). Ann. Bot..

[43] Lillo C. (1994). Light regulation of nitrate reductase in green leaves of higher plants. Physiol. Plant..

[44] Cheng C.L., Acedo G.N., Cristinsin M., Conkling M.A. (1992). Sucrose mimics the light induction of arabidopsis nitrate reductase gene transcription. Proc. Natl. Acad. Sci. USA.

[45] Sivasankar S., Rothstein S., Oaks A. (1997). Regulation of the accumulation and reduction of nitrate by nitrogen and carbon metabolites in maize seedlings. Plant Physiol..

[46] Blom-Zandstra M., Lampe J.E. (1985). The role of nitrate in the osmoregulation of lettuce (*Lactuca sativa* L.) grown at different light intensities. J. Exp. Bot..

[47] Deng J.-M., Bin J.-H., Pan R.-C. (2000). Effects of light quality on the primary nitrogen assimination of rice (*Oryza sativa* L.) seedlings. Acta Bot. Sin..

[48] Shi H., Han J., Guan C., Yuan T. (1999). Effects of red and blue light proportion on leaf growth, carbon-nitrogen metabolism and quality in tobacc. Acta Agron. Sin..

[49] Lin K.-H., Huang M.-Y., Huang W.-D., Hsu M.-H., Yang Z.-W., Yang C.-M. (2013). The effects of red, blue, and white light-emitting diodes on the growth, development, and edible quality of hydroponically grown lettuce (*Lactuca sativa* L. var. capitata). Sci. Hortic..

[50] Ohashi-Kaneko K., Matsuda R., Goto E., Fujiwara K., Kurata K. (2006). Growth of rice plants under red light with or without supplemental blue light. Soil Sci. Plant Nutr..

[51] Yorio N.C., Goins G.D., Kagie H.R., Wheeler R.M., Sager J.C. (2001). Improving spinach, radish, and lettuce growth under red light-emitting diodes (LEDs) with blue light supplementation. HortScience.

[52] Matsuda R., Ohashi-Kaneko K., Fujiwara K., Goto E., Kurata K. (2004). Photosynthetic characteristics of rice leaves grown under red light with or without supplemental blue light. Plant Cell Physiol..

[53] Duthie G.G., Duthie S.J., Kyle J.A.M. (2000). Plant polyphenols in cancer and heart disease: Implications as nutritional antioxidants. Nutr. Res. Rev..

[54] Pech R., Volna A., Hunt L., Bartas M., Cerven J., Pecinka P., Spunda V., Nezval J. (2022). Regulation of phenolic compound production by light varying in spectral quality and total irradiance. Int. J. Mol. Sci..

[55] Rosati C., Cadic A., Duron M., Ingouff M., Simoneau P. (1999). Molecular characterization of the anthocyanidin synthase gene in Forsythia×intermedia reveals organ-specific expression during flower development. Plant Sci..

[56] Ma X., Ma H., Chen Q., Ma Y., Daugulis A.J., Liang J., Zheng P. (2021). The Influence of monochromatic lights on flavonoid production by the fungus sanghuangporus vaninii: Modeling of kinetic profiles and expression levels of important genes in flavonoid synthesis. Biochem. Eng. J..

[57] Dou H., Niu G., Gu M. (2019). Photosynthesis, morphology, yield, and phytochemical accumulation in basil plants influenced by substituting green light for partial red and/or blue light. HortScience.

[58] Miao C., Yang S., Xu J., Wang H., Zhang Y., Cui J., Zhang H., Jin H., Lu P., He L. (2023). Effects of light intensity on growth and quality of lettuce and spinach cultivars in a plant factory. Plants.

[59] Shimizu H., Saito Y., Nakashima H., Miyasaka J., Ohdoi K. (2011). Light environment optimization for lettuce growth in plant factory. IFAC Proc. Vol..

[60] Lee J.H., Kwon Y.B., Roh Y.H., Choi I.L., Kim J., Kim Y., Yoon H.S., Kang H.M. (2023). Effect of various LED light qualities, including wide red spectrum-LED, on the growth and quality of mini red romaine lettuce (*cv. Breen*). Plants.

[61] Razzak M.A., Asaduzzaman M., Tanaka H., Asao T. (2022). Effects of supplementing green light to red and blue light on the growth and yield of lettuce in plant factories. Sci. Hortic..

[62] Kim H.-H., Goins G.D., Wheeler R.M., Sager J.C. (2004). Green-light supplementation for enhanced lettuce growth under red- and blue-light-emitting diodes. HortScience.

